# Fraction-dependent dose dynamics and clinical safety in high-risk neuroblastoma treated with [^177^Lu]Lu-DOTATATE: results from the LuDO-N trial

**DOI:** 10.1007/s00259-026-07984-2

**Published:** 2026-06-19

**Authors:** Se Whee Sammy Park, Fredrik Sundquist, Leonor Teles, Rob van Rooij, Daniel Thor, Kasper Karlsson, Arthur J.A.T. Braat, Jakob Stenman, Joachim N. Nilsson

**Affiliations:** 1 Present Address: Department of Oncology-Pathology, Karolinska Institutet, Stockholm, Sweden; 2 Department of Urology, Karolinska University Hospital, Stockholm, Sweden; 3 Department of Women’s and Children’s Health, Karolinska Institutet, Stockholm, Sweden; 4 Princess Maxima Center for Pediatric Oncology, Utrecht, The Netherlands; 5 Department of Radiology and Nuclear Medicine, University Medical Center, Utrecht, The Netherlands; 6 Department of Nuclear Medicine and Medical Radiation Physics, Karolinska University Hospital, Stockholm, Sweden; 7 Department of Pediatric Surgery and ECMO Center, Karolinska University Hospital, Stockholm, Sweden; 8 Department of Molecular Medicine and Surgery, Karolinska Institutet, Stockholm, Sweden

**Keywords:** LuDO-N trial, ^177^Lu-DOTATATE, High-risk neuroblastoma, Molecular radiotherapy, Dosimetry, Clinical safety

## Abstract

**Purpose:**

Neuroblastoma frequently overexpresses somatostatin receptors (SSTR), enabling molecular radiotherapy with [^177^Lu]Lu-DOTATATE (^177^Lu-DOTATATE). Based on prior studies suggesting that dose intensification is required to achieve objective responses, the LuDO-N trial employs a two-fraction, high-activity treatment regimen. This study aimed to evaluate fraction-dependent changes in tumour and risk-organ absorbed doses, as well as the clinical safety of the intensified ^177^Lu-DOTATATE treatment approach.

**Methods:**

Fourteen patients with high-risk recurrent or refractory neuroblastoma received ^177^Lu-DOTATATE in a dose-intensified, dosimetry-guided regimen. Treatment was given in two fractions 2–3 weeks apart, targeting a cumulative dose of either 2.4 Gy to the whole body or 23 Gy to either kidney. The first fraction was administered at 200 MBq/kg, while the second fraction was individually adjusted to achieve the dosimetric targets.

**Results:**

Tumour-to-kidney and tumour-to-whole-body absorbed dose ratios both decreased significantly by 44% after the second fraction, compared to the first. Tumours received a median 53% lower absorbed dose per administered activity in the second fraction, while kidney and whole-body doses per activity were reduced by 24% and 18%, respectively. Four of fourteen patients (29%) underwent peripheral blood stem cell reinfusion. No sustained renal toxicity was observed.

**Conclusions:**

These findings support a front-loaded ^177^Lu-DOTATATE strategy to maximise tumour irradiation while maintaining exposure to risk organs within safety limits. Accordingly, the LuDO-N protocol has been amended to increase administered activity to 400 MBq/kg in the first fraction for subsequent patients. Together, this work contributes to the ongoing optimisation of dosimetry-guided and intensified treatment strategies for SSTR-targeted molecular radiotherapy for high-risk neuroblastoma.

**Clinical trial registration:**

EU Clinical Trials Register, EU CT 2023-503684-42-00. Registered 2021-05-21.

https://euclinicaltrials.eu/search-for-clinical-trials/?lang=en&EUCT=2023-503684-42-00.

**Supplementary Information:**

The online version contains supplementary material available at https://doi.org/10.1007/s00259-026-07984-2.

## Introduction

Neuroblastoma (NB) is a paediatric tumour that can be stratified into low-, intermediate- and high-risk NB [1]. Patients with high-risk NB that is refractory to standard first and second line therapies, or who experience a relapse, have limited treatment options and a poor prognosis [2, 3]. A recent review by Pawne et al. [4] highlighted the growing potential of molecular radiotherapy (MRT) as a promising approach for this challenging patient population. One such approach involves the use of somatostatin receptor (SSTR)-targeted MRT such as [^177^Lu]Lu-DOTATATE (^177^Lu-DOTATATE), for patients with tumours exhibiting high SSTR expression.

^177^Lu-DOTATATE has been evaluated in several paediatric clinical settings. In the NETTER-P trial (NCT04711135), eleven patients with neuroendocrine tumours received up to four fractions of 7.4 GBq/cycle at 8–12 weeks intervals, demonstrating the feasibility and safety of administering ^177^Lu-DOTATATE to paediatric patients [5]. In refractory or recurrent high-risk NB, the phase IIa LuDO trial treated eight patients with four fractions of 75–100 MBq/kg given 8–12 weeks apart [6]. However, despite high tumour avidity, clinical responses were limited, possibly due to insufficient treatment intensity for this rapidly progressing disease [6]. In response, the ongoing phase II LuDO-N trial (NCT04903899) implements an intensified regimen of two fractions spaced 2–3 weeks apart [7, 8]. In the first fraction, patients received 200 MBq/kg followed by a dosimetry-guided “top-up” to reach either a cumulative renal dose of 23 Gy–2.4 Gy to the whole-body, which serves as a proxy for bone marrow exposure [7]. In parallel, the phase I NEUROBLU 02 trial (NCT03966651) is evaluating a dose-escalation regimen of two fractions of 80–120 MBq/kg administered six weeks apart.

The previous LuDO trial also reported a substantial decrease in tumour affinity over treatment cycles [9]. This amounted to approximately three-fold lower absorbed dose delivered to tumours per administered activity in the fourth vs. first administration. Furthermore, no objective radiological response was observed, indicating that the reduction in tumour uptake was most likely due to SSTR downregulation rather than treatment response. Similar trends have also been observed in adult patients with neuroendocrine tumours, where tumour uptake decreased across successive ^177^Lu-DOTATATE administrations [10–14].

In this study, tumour and risk organs dosimetry were analysed for the first 14 patients treated within the LuDO-N trial. Additionally, longitudinal haematological and renal parameters were evaluated to assess toxicity. Together, these analyses provide insight into dose dynamics over multiple administrations and clinical safety under the intensified treatment regimen, supporting further optimisation for ^177^Lu-DOTATATE scheduling for high-risk NB.

## Materials and methods

### Study design and patients

The phase II, multicentre, single-arm, open-label LuDO-N trial (EuCT 2023-503684-42-00; NCT04903899) is open in Scandinavia, the Netherlands, Lithuania, the United Kingdom and Spain. Between 2021 and 2025, 14 patients with relapsed or refractory high-risk NB were enrolled (Table 1). Seven patients were treated at Karolinska University Hospital, Stockholm, Sweden, and seven at the Princess Máxima Center for Pediatric Oncology, Utrecht, the Netherlands.

**Table 1:** Patient characteristics and ^177^Lu-DOTATATATE administered activity

Patients (*n* = 14)			
Age at registration, median (range) [years]	7.6 (2.4–15.1)		
Sex, Male: Female (%)	6 (43%): 8 (57%)		
Weight at treatment, median (range) [kg]	21 (14–61)		
**Disease status**			
Refractory	5 (36%)		
Relapsed	9 (64%)		
**Primary site**			
Abdomen	13 (93%)		
Bone	1 (7%)		
**Tumour site involvement**			
Primary tumour	5 (36%)		
Bone marrow metastases	8 (57%)		
Lymph node metastases	2 (14%)		
**Prior treatment**			
Surgery	9 (64%)		
External beam radiotherapy	9 (64%)		
High dose chemotherapy	10 (71%)		
**Genetic alterations**			
MYCN amplification	1 (7%)		
11q deletion	10 (71%)		
TERT rearrangement	1 (7%)		
^177^ **Lu-DOTATATE administered activity**	**Fraction 1**	**Fraction 2**	**Total**
Median (IQR) [GBq]	4.3 (3.6-6.0)	8.5 (5.8–10.9)	13.5 (9.8–15.6)
Weight-adjusted – Median (IQR) [MBq/kg]	208 (197–219)	407 (297–558)	610 (496–788)

### ^177^Lu-DOTATATE treatment

Antiemetics were administered 1 h before treatment. A concomitant arginine/lysine solution (LysaKare^®^) was infused starting 30 min before treatment and continued for up to 4 h. Patients received intravenous hydration 6 h before and continuing until 18 h after treatment. ^177^Lu-DOTATATE (Lutathera^®^) was administered intravenously over 20–60 min. For the first treatment fraction, patients received 200 MBq/kg. The activity administered in the second fraction was determined by aiming for a cumulative absorbed dose of 2.4 Gy to the whole body or 23 Gy to either kidney, whichever limit was reached first. The two fractions were administered 2–3 weeks apart. All patients had a minimum of 2 × 10^6^ peripheral blood stem cells (PBSC) as a back-up, in case of a prolonged treatment-related myelotoxicity.

### PET/CT and SPECT/CT imaging

Prior to treatment, patients were screened using [^68^Ga]Ga-DOTATOC (SomaKit TOC^®^) PET/CT to assess trial eligibility. The PET/CT acquisitions were not standardised within the trial and were performed according to local protocols at the respective referring centres. Images were acquired at approximately 60 min post-injection and uptake of [^68^Ga]Ga-DOTATOC had to be greater in the tumour lesions than in the liver for eligibility. After each ^177^Lu-DOTATATE administration, whole-body imaging was performed at 0–4 h, 1 day, 2–5 days and 6–9 days post-infusion. This was combined with SPECT/CT at the three later timepoints (Suppl. Materials and Methods).

### Tumour, kidney and whole-body dosimetry

Dosimetry was performed using serial post-therapeutic whole-body imaging to estimate whole-body absorbed dose and SPECT/CT imaging to estimate tumour and kidney doses. [^68^Ga]Ga-DOTATOC PET/CT was used to guide lesion selection. In patients with evaluable tumour lesions, one to three lesions were analysed, and the arithmetic mean was used in case of multiple lesions. Kidney absorbed doses were based on the mean of both kidneys, except in one patient who had previously undergone unilateral nephrectomy. An experienced nuclear medicine physicist responsible for dosimetry at the Karolinska site performed the dosimetry together with an experience nuclear medicine physician. Calculations were performed using an in-house software for the conversion from cps to dose rate and subsequent time integration.

Tumour dose comparisons were based on the maximum tumour ^177^Lu-DOTATATE activity concentration measured within each lesion at all SPECT/CT timepoints. Maximum activity concentration can serve as a reproducible proxy for tumour absorbed doses as tumour volume and signal spill-over is assumed to remain largely consistent during the relatively short interval between fractions. To validate the assumption of non-changing tumour volumes between fractions, treatment response at one-month post-administration was assessed. The maximum tumour activity concentrations were integrated over time using monoexponential fitting, resulting in time-integrated activity concentration values with units [MBq*h/ml]. Using this quantity, tumour dose ratios between the two treatment fractions were calculated for each patient. This approach was chosen over volume-based absorbed dose calculations as many patients had small bone marrow metastases for which reliable volume delineation was not possible.

Whole-body doses were calculated using a monoexponential fit to total body activity content, derived from geometric means of the anterior and posterior scans. The activity content was integrated over time and multiplied with an age-adjusted S-value of whole-body to whole-body (OLINDA version 2.1). Kidney dosimetry was performed using an activity-concentration approach, with 1–5 small region-of-interests (ROIs; 0.6 ml each) placed in central regions of the renal cortex. Activity concentrations in kidneys were converted to dose rate using a factor invariant of kidney size, similar to previously described methodology [15]. The factor was 0.0875 Gy/(MBq*h/ml), calculated through in-house Monte Carlo N-Particle (MCNP) simulations. The resulting dose rates were subsequently integrated over time into absorbed dose.

### Dosimetry analysis

Changes in tumour-to-kidney (Eq. 1) and tumour-to-whole-body (Eq. 2) dose ratios were expressed as a percentage change in fraction 2 relative to fraction 1 (F2/F1). For each fraction, the dose ratios were calculated as maximum tumour activity concentration divided by either mean kidney or whole-body absorbed dose.

1 $$\mathrm{Change}\;\mathrm{in}\;\mathrm{tumour}\mathrm{-}\mathrm{to}\mathrm{-}\mathrm{kidney}\;\mathrm{ratio}\;\left(\%\right)=\frac{\mathrm{Tumour}\mathrm{-}\mathrm{to}\mathrm{-}\mathrm{kidney}\;{\mathrm{ratio}}_{\mathrm F2}}{\mathrm{Tumour}\mathrm{-}\mathrm{to}\mathrm{-}\mathrm{kidney}\;{\mathrm{ratio}}_{\mathrm F1}}\cdot\;100$$

2 $$\mathrm{Change}\;\mathrm{in}\;\mathrm{tumour}\mathrm{-}\mathrm{to}\mathrm{-}\mathrm{whole}\mathrm{-}\mathrm{body}\;\mathrm{ratio}\;\left(\%\right)=\frac{\mathrm{Tumour}\mathrm{-}\mathrm{to}\mathrm{-}\mathrm{whole}\mathrm{-}\mathrm{body}\;{\mathrm{ratio}}_{\mathrm F2}}{\mathrm{Tumour}\mathrm{-}\mathrm{to}\mathrm{-}\mathrm{whole}\mathrm{-}\mathrm{body}\;{\mathrm{ratio}}_{\mathrm F1}}\cdot\;100$$

Changes in dose per administered activity to the tumours, kidney and whole-body after each ^177^Lu-DOTATATE administration were calculated as a percentage change in F2 relative to F1 (Eq. 3). For kidneys and whole-body, absorbed dose was normalised to administered activity, whereas for tumour lesions, maximum activity concentration was normalised to administered activity.

3 $$\mathrm{Change}\;\mathrm{in}\;\mathrm{Dose}/\mathrm{Activity}\;(\%)=\frac{\mathrm{Dose}\;\mathrm{per}\;\mathrm{administered}\;{\mathrm{activity}}_{\mathrm F2}}{\mathrm{Dose}\;\mathrm{per}\;\mathrm{administered}\;{\mathrm{activity}}_{\mathrm F1}}\cdot\;100$$

### Clinical safety analysis

Blood samples for platelets, haemoglobin, white blood cells, neutrophils, lymphocytes and creatinine were collected just before administration of the first fraction of ^177^Lu-DOTATATE (considered baseline). Subsequently, samples were drawn after the first fraction (3–6 days after), just before and then after the second fraction (again, 3–6 days after), and subsequently at follow-up visits after end of treatment. Data were analysed up to 6 months after end of treatment.

### Statistical analysis

Comparison of tumour-to-kidney and tumour to whole-body ratio across treatment fractions were performed using the non-parametric Wilcoxon rank-sum test. Associations between PBSC reinfusion status and total administered activity normalised to body weight, whole-body absorbed dose normalised to administered activity and change in nadir from baseline for haematological and renal parameters were also evaluated using the Wilcoxon rank-sum test. Paired comparisons of absorbed dose per ^177^Lu-DOTATATE administration between treatment fractions were analysed using the non-parametric Wilcoxon signed-rank test. Confidence intervals for the median were estimated using bootstrapping. All statistical analysis was performed using R (version 4.5.2).

## Results

### Patients and treatments

All patients received two administrations of ^177^Lu-DOTATATE. At the first fraction, a median of 4.3 GBq (IQR 3.6–6.0 GBq) activity was administered, corresponding to 208 MBq/kg (IQR 197–219 MBq/kg), in accordance with the treatment protocol of 200 MBq/kg (Table 1). The second administration was individually adjusted to reach renal or whole-body dosimetric targets, resulting in a median activity of 8.5 GBq (IQR 6.0-10.9 GBq; median 407 MBq/kg, IQR 297–558 MBq/kg; Table 1), for a median cumulative dose of 610 MBq/kg (IQR 496–788 MBq/kg). The median cumulative absorbed dose was 16 Gy (IQR 15–18 Gy) to kidneys and 1.2 Gy (IQR 1.0–1.4 Gy) to whole-body (Table 2). No patients exceeded the kidneys nor whole-body dose thresholds. One patient was excluded from the dosimetric analyses due to pre-existing hydronephrosis that required ureteric stenting to avoid confounding effects (Table 2). Furthermore, tumour lesion analysis was not performed in three patients due to diffuse bone marrow involvement with low uptake relative to background signal at both treatment fractions. As the lesions could not be reliably followed over time, neither anatomically nor functionally, even when using the maximum activity concentration approach, these patients were excluded from the tumour comparisons to avoid introducing unreliable data points.

**Table 2:** ^177^Lu-DOTATATE administered activity, kidney and whole-body dosimetry and effective half-life (T_eff_) for kidney for all trial participants across treatment fractions. Kidney doses and effective half-life represent the mean across both kidneys, except for the patient who underwent a unilateral nephrectomy prior to trial enrollment

Patient	Frac.	Activity [GBq]	Activity per body weight [MBq/kg]	Kidney dose [Gy]	Total kidney dose [Gy]	Whole-body dose [Gy]	Total whole-body dose [Gy]	Kidney T_eff_ [hrs]	$\:\varDelta\:$Kidney T_eff_ [hrs]
1	1	3.1	220	8	19	0.6	1.6	63	18
	2	5.7	413	11		1.0		45	
2	1	4.1	230	5	15	0.5	1.3	51	5
	2	10.6	589	10		0.8		46	
3	1	13.3	217	6	18	0.4	1.3	62	12
	2	29.3	494	11		0.9		50	
4	1	4.9	209	6	18	0.4	1.3	49	3
	2	15.1	659	12		0.9		46	
5^#^	1	4.3	213	8	20	0.5	1.3	67	9
	2	8.1	401	12		0.8		58	
6	1	3.6	221	8	23	0.4	1.4	59	13
	2	11.5	701	15		1.0		46	
7	1	3.1	235	6	15	0.7	1.6	62	16
	2	7.7	579	9		0.9		46	
8	1	6.2	206	7	16	0.4	1.0	62	3
	2	11	357	9		0.6		59	
9	1	3	194	6	14	0.4	1.1	48	1
	2	6.4	413	8		0.7		47	
10	1	5.3	202	6	18	0.4	0.9	47	7
	2	8.9	324	12		0.5		40	
11	1	6.4	198	5	12	0.4	1.0	54	-2
	2	9.4	288	7		0.6		56	
12	1	3.6	196	6	15	0.5	1.1	58	-6
	2	4.5	241	9		0.6		64	
13*	1	4.2	197	8	13	0.6	0.9	64	-8
	2	1.5	72	5		0.3		72	
14	1	6.9	195	8	16	0.5	0.8	57	-5
	2	5.8	158	8		0.3		62	

*Hydronephrosis diagnosis prior to and during treatment, not included in dosimetry nor kidney T_eff_ analysis. ^#^Unilateral nephrectomy prior to treatment. Frac. = treatment fraction; hrs = hours

### Tumour and organ dosimetry

**Fig. 1:**
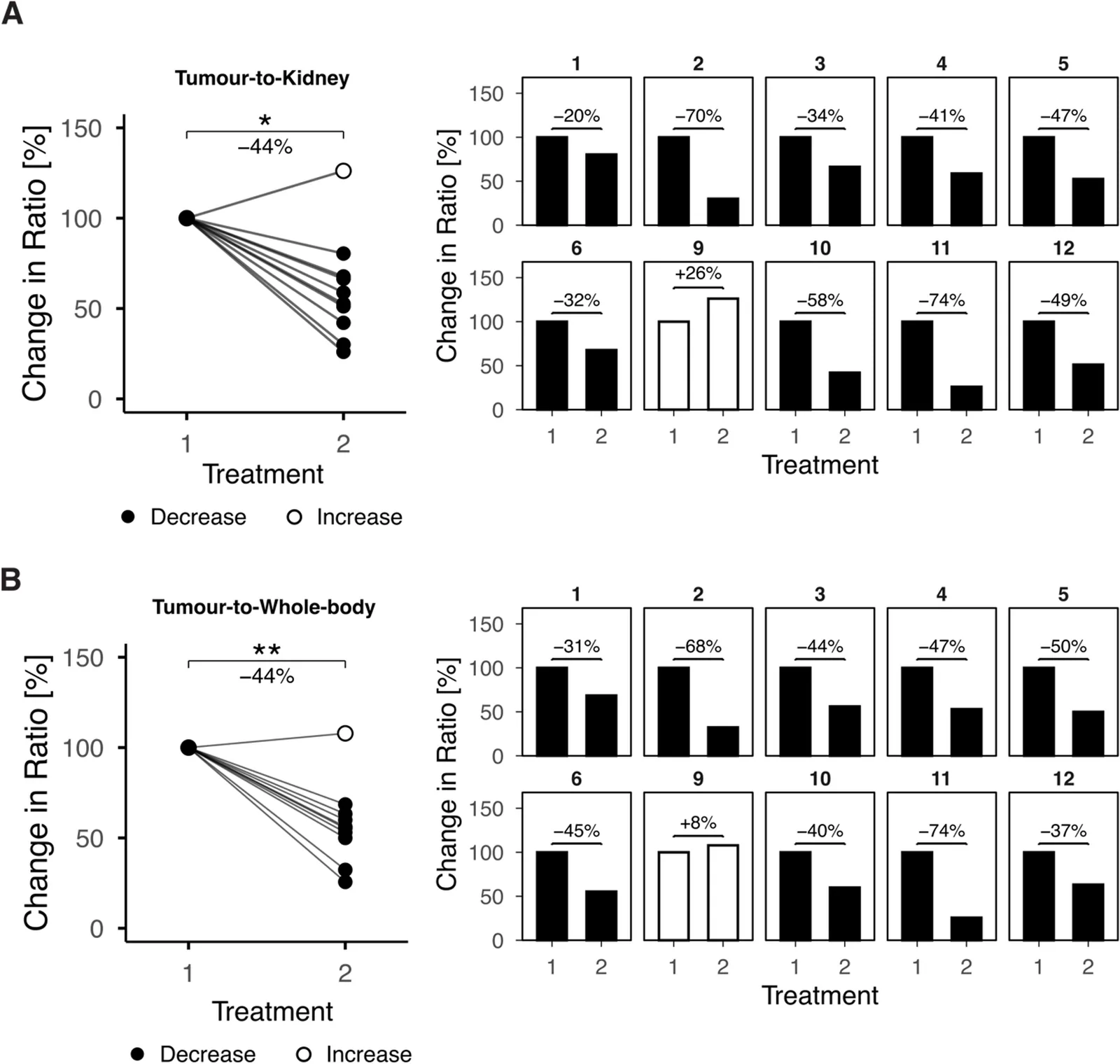
Reduced therapeutic ratio between the first and second administration of ^177^Lu-DOTATATE. **A** | Tumour-to-kidney absorbed dose ratios for the two treatment administrations. Lines connect paired patient values. Bar plots depict individual percentage changes in tumour-to-kidney ratio for each patient (numbers corresponding to Table 2), normalised to the first treatment fraction. **B** | Tumour-to–whole-body absorbed-dose ratios. Plot structure and statistical analysis are identical to (A). Across all panels, filled shapes indicate a decrease in ratio, whereas unfilled shapes indicate an increase. In the left panels, median change in ratio is shown; significance levels are as indicated: * (*p* ≤ 0.05), ** (*p* ≤ 0.01)

The tumour-to-kidney and tumour-to-whole-body dose ratios were significantly reduced in the second fraction of ^177^Lu-DOTATATE compared with the first. The tumour-to-kidney dose ratio decreased by a median of 44% (95% CI 27–59%) relative to fraction 1, with values ranging from a 74% reduction to a 26% increase (Fig. 1A). Similarly, the tumour-to-whole-body ratio decreased by a median of 44% (CI 36–57%; range − 74% to 8%; Fig. 1B). All patients showed a reduction in both ratios except for one patient who had an increase of 26% and 8% in the tumour-to-kidney and tumour-to-whole body ratios, respectively. This patient also had among the lowest kidney doses but did not differ in any other obvious way (Table 2). Changes in the therapeutic ratios (Suppl. Figure 1A) were comparable between centres, indicating agreement in imaging and dosimetric methodology (Suppl. Figure 1A).

**Fig. 2:**
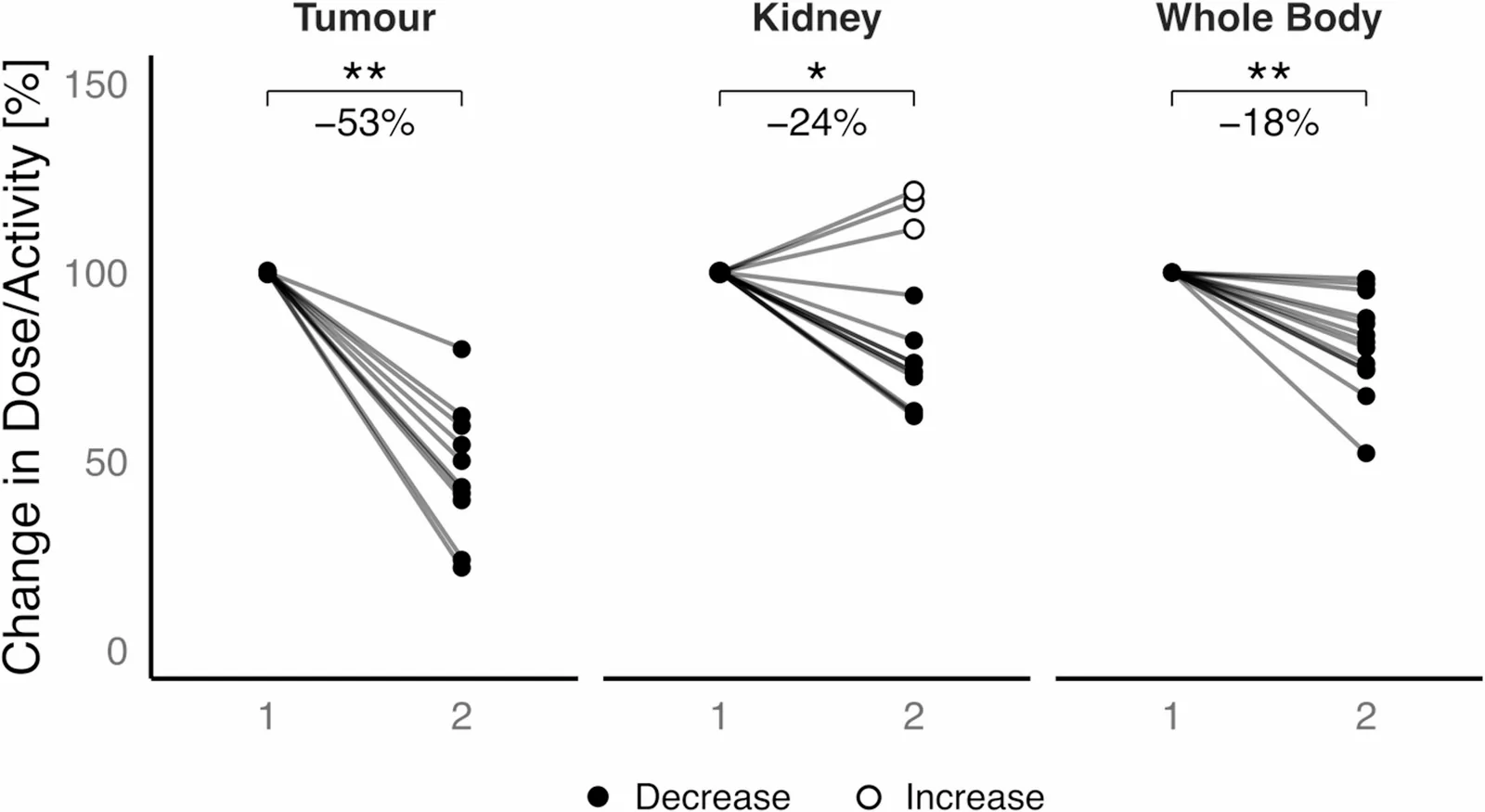
Reduced absorbed doses between the first and second ¹⁷⁷Lu-DOTATATE administrations. **A** | Absorbed dose per administered activity for tumour lesions, kidneys, and whole body in the first and second fractions, normalised to the first fraction. Lines connect paired patient values. Filled shapes indicate a decrease in ratio, whereas unfilled shapes indicate an increase. Median change in activity-normalised dose is shown. Significance levels: ns (*p* > 0.05), * (*p* ≤ 0.05), ** (*p* ≤ 0.01)

Absorbed doses per administered activity of ^177^Lu-DOTATATE were consistently lower in the second fraction across tumours, kidneys, and whole-body (Fig. 2). Tumour absorbed dose per activity showed the greatest reduction, with a median decrease of 53% (CI 41–60%). The reductions in kidneys and whole-body were smaller but still statistically significant at 24% (CI 6–28%) and 18% (CI 12–26%), respectively. Most patients had consistent reductions, however three patients exhibited higher renal absorbed doses in the second fraction (Fig. 2). The reduction in kidney absorbed doses was driven in part by a shorter effective half-life of ^177^Lu-DOTATATE in the kidneys during the second fraction, corresponding to an average decrease of 6 h, or about 10% (Table 2). Furthermore, absorbed doses for kidneys and whole-body did not differ between the two treatment centres, again indicating no discrepancy in methodology (Suppl. Figure 1B).

**Fig. 3:**
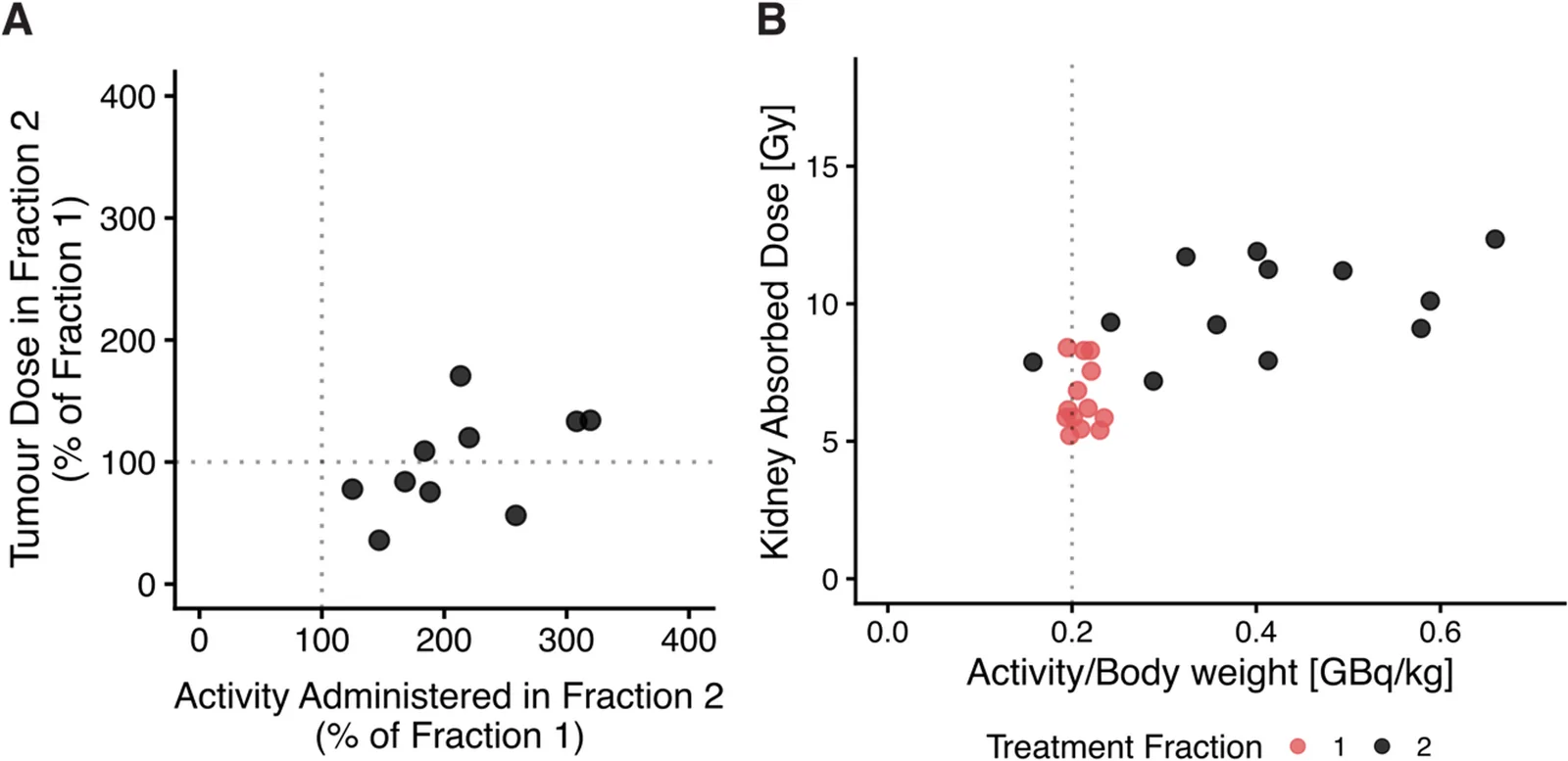
Administered activity of ^177^Lu-DOTATATE and tumour and kidney dose. **A** | Percentage change in administered activity in fraction 2 compared to fraction 1 and the corresponding change in tumour dose for all patients with evaluable tumour lesions. The vertical dotted line indicates equal administered activity in fraction 1 and 2. The horizontal dotted line indicates equal dose in fraction 1 and 2. **B** | Administered activity and kidney absorbed doses at both fractions. The red dots indicate the first treatment fraction, where a fixed activity of approximately 0.2 GBq/kg (dotted lines) was administered to all patients

As higher administered activities also mean increased amounts of peptide delivered, potentially leading to receptor saturation, we explored whether greater activity resulted in a plateau in tumour and kidney doses. For tumours, the change in administered activity between fractions was plotted against the corresponding change in tumour dose (Fig. 3A). Visual inspection did not indicate an apparent plateau and higher administered activity generally coincided with higher tumour concentrations. Despite all patients receiving higher administrations of activity in the second fraction, all tumour concentrations remained lower than expected. For kidneys, administered activity and absorbed doses from both fractions were plotted (Fig. 3B). Kidney absorbed doses also seemed to increase with administered activity and no apparent plateau was observed.

### Clinical safety

**Fig. 4:**
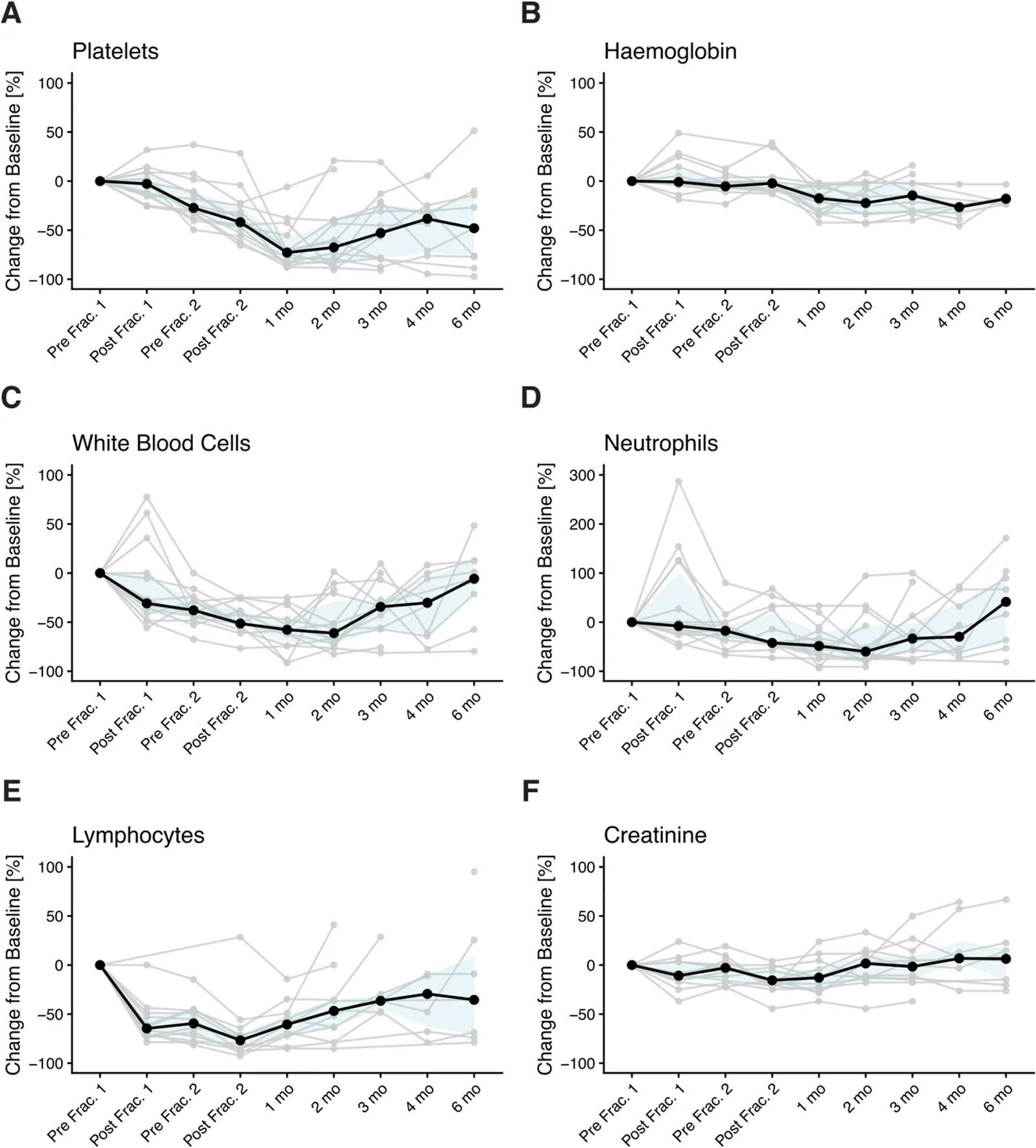
Longitudinal trajectories of haematological and renal parameters during and after ^177^Lu-DOTATATE treatment. Relative changes from baseline (%) are shown for (**A**) platelets, (**B**) haemoglobin, (**C**) while blood cells, (**D**) neutrophils, (**E**) lymphocytes and (**F**) creatinine. Baseline is defined as the measurements taken in the first fraction of ^177^Lu-DOTATATE (Pre Frac. 1). Each grey line represents an individual patient trajectory. The black line represents the median, with the blue-ribbon indicating IQR

Longitudinal trajectories of haematological and renal parameters were assessed during and after ^177^Lu-DOTATATE treatment for all patients (Fig. 4, Suppl. Figure 2). Although longer follow-up was available for some patients, analyses were limited to the first six months after treatment, corresponding to a complete follow-up for more than 50% of patients. For the analysis, all parameters were normalised to each patient’s value collected right before the first administration of ^177^Lu-DOTATATE; if unavailable, the most recent prior measurement was used as baseline. Haemoglobin values taken in connection with erythrocyte transfusions were excluded from analysis. One patient’s lymphocyte value was unavailable, and another patient was excluded from the creatinine analysis due to pre-existing hydronephrosis. One month after completion of treatment, other therapies with potential haematological toxicity were administered for most patients. Therefore, haematological changes observed beyond this point cannot be attributed solely to ¹⁷⁷Lu-DOTATATE and should be interpreted with caution.

Platelet counts declined following treatment, reaching a median relative change from baseline of − 73% (IQR − 83% to − 59%) at one month after end of treatment, followed by a progressive recovery (Fig. 4A, Suppl. Table 1). The corresponding median absolute platelet levels were 44 × 10^9^/L (IQR 30–72 × 10^9^/L) at one month (Suppl. Figure 2 A). Haemoglobin showed a nadir at four months with a median relative change of − 27% (IQR − 34% to − 25%) and median absolute value of 85 g/L (IQR 84–90 g/L; Fig. 4B, Suppl. Figure 2B). White blood cell counts reached a nadir at two months with median relative decrease of − 61% (IQR − 72% to − 29%), while the lowest absolute median value was at one month (2 × 10^9^/L, IQR 1–3 × 10^9^/L), followed by a recovery at six months (Fig. 4C, Suppl. Figure 2 C). Similarly, neutrophils reached their lowest relative suppression at two months (− 60%, IQR − 77% to − 7%; 1 × 10^9^/L, IQR 0.6-2 × 10^9^/L) followed by a recovery to baseline (Fig. 4D, Suppl. Figure 2D). Lymphocytes showed the greatest relative suppression after the second administration (− 77%, IQR − 86% to − 71%), mirrored by an absolute median nadir of 0.3 × 10^9^/L (IQR 0.2–0.5 × 10^9^/L; Fig. 4E, Suppl. Figure 2E). In contrast, creatinine levels remained relatively stable, with median peak of 7% (IQR 3% to 24%; 34 µmol/L, IQR 27–39 µmol/L) at four months (Fig. 4F, Suppl. Figure 2 F).

**Fig. 5:**
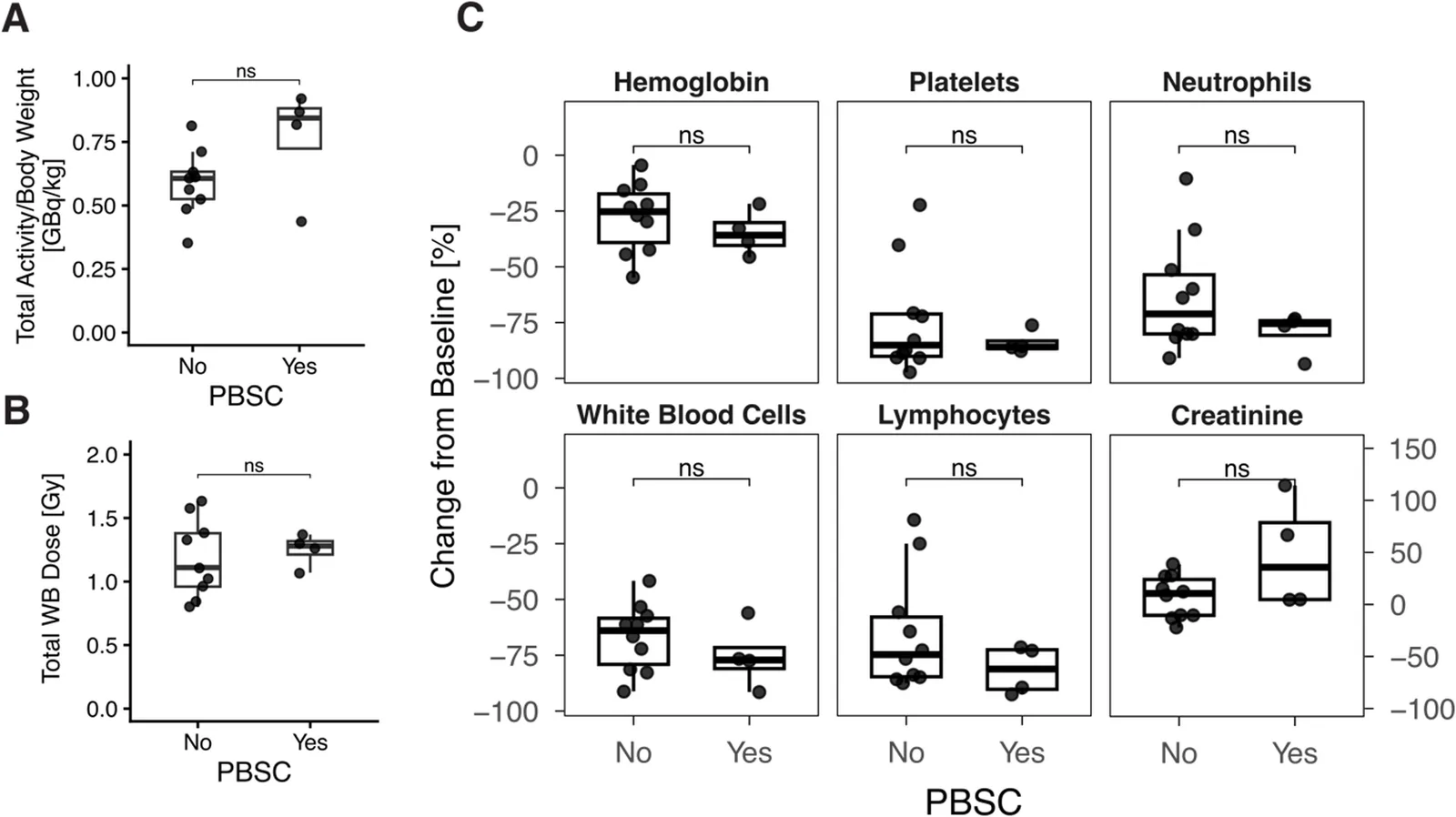
Administered ^177^Lu-DOTATATE activity, whole-body dose and haematological parameters in patients who have or have not received peripheral blood stem cell (PBSC) reinfusion. **A** | Total administered activity of ^177^Lu-DOTATATE per body weight, stratified by patients who have or have not undergone PBSC reinfusion. **B |** Total whole-body absorbed dose between the same patient stratifications as (A). **C |** Change (%) from baseline to nadir for haematological parameters and change (%) from baseline to peak for creatinine, stratified as (A). Significance levels: ns (*p* > 0.05)

Four out of fourteen treated patients underwent PBSC reinfusion. In two of those patients, reinfusion occurred about 10 weeks after the end of treatment. One patient received reinfusion by around 5–6 weeks due to follow-up treatments. For the remaining patient, the date of reinfusion was not available. Patients receiving PBSC reinfusion did not receive significantly higher administrations of ^177^Lu-DOTATATE compared with those who did not (Fig. 5A). Similarly, cumulative whole-body absorbed dose did not differ between patients who did and did not receive PBSC reinfusion (Fig. 5B). No significant differences were observed in the percentage decrease from baseline to nadir for haematological or renal parameters (Fig. 5C).

## Discussion

In this study, tumour, kidney and whole-body dosimetry as well as haematological and renal parameters were evaluated after the sequential administrations of ^177^Lu-DOTATATE to assess the therapeutic and clinical safety implications of the intensified dosing regimen in the LuDO-N trial.

The second fraction of ^177^Lu-DOTATATE delivered a significantly lower dose per activity to the tumour in comparison to the kidney or whole-body than in the first (Fig. 1). No evidence of tumour saturation was observed, as increasing administered activity was not associated with a plateau in tumour dose (Fig. 3A). Similar reductions in tumour uptake across sequential treatments were also reported in the LuDO trial [9]. Although the underlying mechanisms remains unclear, several explanations may be considered. SSTR2 downregulation could reduce tumour uptake. However, given that SSTR recycling in neuroendocrine tumours occurs within hours, persistent downregulation after a two-week interval between fractions seems unlikely [16–18]. A treatment-induced reduction in tumour volume could also contribute, but the absence of substantial radiological changes at one-month follow-up (unpublished data) makes tumour shrinkage a less likely primary explanation. Other possible explanations may include radiation-induced changes within the tumour microenvironment, potentially limiting ^177^Lu-DOTATATE accumulation in the second fraction.

Kidney and whole-body absorbed doses also declined in the second fraction (Fig. 2). As dosimetric targets were based on extrapolations from the first fraction, this reduction contributed to the fact that the total targets were not reached in most patients. This observation contrasts with previous reports in which reduced tumour uptake was associated with increased renal exposure due to higher levels of circulating activity [19, 20]. The reduction could not be explained by receptor saturation. Another explanation may be radiation-induced damage to the proximal tubules, potentially reducing peptide reabsorption capacity, resulting in faster clearance in the second fraction. This interpretation is supported by the approximately 10% shorter renal effective half-life (Table 1). In contrast, adult neuroendocrine tumour studies have generally reported stable kidney doses across treatments [13, 21], possibly due to longer intervals between treatment (8–12 weeks) [22] compared with this trial (2–3 weeks), allowing greater renal recovery between administrations. However, no clinically meaningful long-term decline in renal function was observed in this study, as reflected by stable creatinine values (Fig. 4F), suggesting that potential radiation-related effects were limited or that alternative mechanisms may contribute to the altered renal kinetics.

Interpretation of haematological toxicities following ¹⁷⁷Lu-DOTATATE in this cohort is inherently challenging. According to the study protocol, patients were allowed to proceed to subsequent therapies as early as one month after completion of ¹⁷⁷Lu-DOTATATE treatment, and most patients did. Consequently, cytopenias after the first month cannot be attributed to ¹⁷⁷Lu-DOTATATE alone and may reflect cumulative effects of subsequent therapies (Fig. 4). Overall, the haematological measurements were consistent with previous reports of MRT-associated bone marrow suppression in heavily pre-treated paediatric patients [6]. Lymphopenia was similarly observed in most patients in the NETTER-1 trial [23]. Despite this, the NETTER-1 trial did not see an increase in clinically significant infections, possibly because ^177^Lu-DOTATATE affect mostly B-cells while sparing T-cells [23], suggesting that the ^177^Lu-DOTATATE-induced lymphopenia may be clinically manageable. Notably, greater cumulative whole-body absorbed doses were not associated with subsequent PBSC reinfusion (Fig. 5), indicating no clear link between bone marrow exposure and clinically significant myelotoxicity within the studied range.

The small sample size is an inherent limitation of this study and results should be interpreted with caution. Exclusions of three patients with difficult-to-delneate tumour lesions may have introduced selection bias. However, we believe inclusion of unreliable tumour dosimetry data would have increased uncertainty and risk misleading tumour comparisons. Furthermore, tumour analyses were based on maximum activity concentrations rather than whole-lesion dosimetry, which may limit absolute accuracy but improves reproducibility. Although no clear change in lesion volume was observed at imaging one month after administration, it cannot be completely ruled out that lesion volumes changed marginally between treatment fractions and could have impacted tumour maximum activity concentration measurements.

Currently established applications of MRT are mainly used for differentiated thyroid cancer and low-grade neuroendocrine tumours, where month-long intervals between treatments are effective in the typically slow-progressing disease. However, more aggressive tumours, such as high-risk NB, will likely require much shorter and intensified treatment regimens. This study indicates that tumour uptake declines after the initial administrations and the findings are consistent with delivering most of the activity early in the treatment course, in a front-loaded strategy, to enhance tumour irradiation without increasing cumulative exposure to organs-at-risk. Notably, patients tolerated a median of 407 MBq/kg in the second fraction (Table 2), double the initial dose, without exceeding risk-organ dosimetric thresholds, supporting the feasibility of this approach. These findings highlight the importance of accounting for fraction-dependent dose dynamics and support the use of imaging- or weight-based dosing to maximise the initial administration while maintaining established safety thresholds.

## Conclusions

In this study, we observed pronounced fraction-dependent changes in tumour and risk organ dosimetry. Tumour uptake of ^177^Lu-DOTATATE was significantly reduced in the second administration, despite higher administered activity, while risk organ doses remained within predefined limits. These findings suggest that redistributing activity to the first fraction may enhance tumour uptake without increasing exposure to the risk organs. Based on this rationale, the LuDO-N trial protocol has been amended to increase the first fraction from 200 MBq/kg to 400 MBq/kg. Further validation of both clinical safety and efficacy will be essential and the results from this amended strategy will inform the continued optimisation of ¹⁷⁷Lu-DOTATATE dosing strategies for patients with relapsed or refractory high-risk NB.

## Supplementary Information

Below is the link to the electronic supplementary material.


Supplementary Material 1



Supplementary Material 2



Supplementary Material 3



Supplementary Material 4

