# Supplementary material for: Fraction-dependent dose dynamics and clinical safety in high-risk neuroblastoma treated with [177Lu]Lu-DOTATATE: results from the LuDO-N trial
Source: Eur J Nucl Med Mol Imaging. 2026 Jun 19;53(11):6398–409. doi: 10.1007/s00259-026-07984-2 (PMC13526122; doi:10.1007/s00259-026-07984-2)
Supplement: Supplementary file 1 [file 259_2026_7984_MOESM1_ESM.docx]

**Supplementary Figure 1. A** | Percentage change in tumour/kidney and tumour/whole-body dose ratios in patients treated at Karolinska University Hospital (KS) and Utrecht Medical Center (UMC). Filled shapes indicate a decrease in ratio, whereas unfilled shapes indicate an increase.  **B** | Absorbed dose per administered activity for kidneys and whole body in patients treated at Karolinska University Hospital (KS) and Utrecht Medical Centre (UMC). Both treatment fractions are shown for each patient. Across panels, statistical comparison was performed using a two-sided Wilcoxon rank-sum test. Box plots show the interquartile range (IQR), median (centre line), and whiskers extending to 1.5 × IQR.
