# Supplementary material for: Fraction-dependent dose dynamics and clinical safety in high-risk neuroblastoma treated with [177Lu]Lu-DOTATATE: results from the LuDO-N trial
Source: Eur J Nucl Med Mol Imaging. 2026 Jun 19;53(11):6398–409. doi: 10.1007/s00259-026-07984-2 (PMC13526122; doi:10.1007/s00259-026-07984-2)
Supplement: Supplementary file 2 [file 259_2026_7984_MOESM2_ESM.docx]

**Supplementary Figure 2. Longitudinal trajectories of blood parameters during and after ^177^Lu-DOTATATE treatment.** Absolute measurements are shown for (A) platelets, (B) haemoglobin, (C) while blood count, (D) neutrophils, (E) lymphocytes and (F) creatinine. Each grey line represents an individual patient trajectory. The black line represents the median, with the blue ribbon indicating IQR.
