# Supplementary material for: Fraction-dependent dose dynamics and clinical safety in high-risk neuroblastoma treated with [177Lu]Lu-DOTATATE: results from the LuDO-N trial
Source: Eur J Nucl Med Mol Imaging. 2026 Jun 19;53(11):6398–409. doi: 10.1007/s00259-026-07984-2 (PMC13526122; doi:10.1007/s00259-026-07984-2)
Supplement: Supplementary file 3 [file 259_2026_7984_MOESM4_ESM.docx]

**Supplementary Materials and Methods**

*SPECT/CT Imaging Details*

**Karolinska University Hospital**

System: Discovery 670 Pro, General Electric Medical Systems

Acquisition: 120 projections, 20 sec dwell time, Medium Energy General Purpose, Step and Shoot

Reconstruction: OSEM 6 iterations / 10 subsets, Butterworth (0.4 cutoff frequency, order 5) with attenuation correction, scatter correction and point spread function deconvolution (Xeleris version 4)

**Princess Maxima Center**

System: Symbia Intevo 16, Siemens Healthineers

Acquisition: 120 projections, 20 sec dwell time, Medium Energy

Reconstruction: OSCGM 20 iterations, 8 subsets, Gauss 10 mm, with attenuation correction and scatter correction
